# An Ad Libitum‐Fed Diet That Matches the Beneficial Lifespan Effects of Caloric Restriction but Acts via Opposite Effects on the Energy‐Splicing Axis

**DOI:** 10.1111/acel.70269

**Published:** 2025-10-21

**Authors:** Amanda E. Brandon, Tamara Pulpitel, Carsten Schmitz‐Peiffer, Lewin Small, Alistair M. Senior, Sophie Stonehouse, Letisha Prescott, Alyssa Face, K. Saiful Islam, Jenny E. Gunton, Jacob George, David Raubenheimer, Gregory J. Cooney, David G. Le Couteur, Stephen J. Simpson

**Affiliations:** ^1^ Charles Perkins Centre The University of Sydney Sydney New South Wales Australia; ^2^ School of Life and Environmental Science The University of Sydney Sydney New South Wales Australia; ^3^ ANZAC Research Institute Concord Hospital and Faculty of Medicine and Health Sydney New South Wales Australia; ^4^ Department of Diabetes and Endocrinology Westmead Hospital Sydney New South Wales Australia; ^5^ Centre for Diabetes, Obesity and Endocrinology Research, Westmead Institute for Medical Research University of Sydney Westmead New South Wales Australia; ^6^ Storr Liver Centre, Westmead Institute for Medical Research Westmead Hospital Sydney New South Wales Australia; ^7^ School of Medical Sciences, Faculty of Medicine and Health The University of Sydney Sydney New South Wales Australia

**Keywords:** ageing, lifespan, liver, nutrition, proteomics

## Abstract

Caloric restriction (CR) with fasting extends lifespan but is difficult to maintain in humans. Here, we compared conventional CR with periods of fasting to an ad libitum‐fed low‐protein, high‐carbohydrate (LPHC) diet diluted 25% with non‐digestible fibre. Both approaches similarly enhanced longevity and metabolic health in mice relative to a control diet. Proteomic analysis of liver tissue revealed that CR increased proteins associated with energy and mitochondrial pathways. By contrast, the LPHC diet reduced these pathways but increased the abundance of proteins associated with RNA metabolism and spliceosome pathways. These results for LPHC support the “energy‐splicing resilience” axis theory of ageing. Our results suggest that ad libitum‐fed diets can be designed to replicate, and potentially enhance, the geroprotective benefits of CR, albeit via different mechanisms, potentially offering a more sustainable dietary approach to longevity extension.

## Graphical abstract

Caloric restriction (CR) and an ad libitum‐fed low‐protein, high‐carbohydrate, high‐fibre (LPHC) diet both improve longevity and metabolic health relative to a control diet. Liver proteomics revealed CR alone increased proteins associated with mitochondrial pathways and energy metabolism. The LPHC diet, however, inhibited CR‐related pathways and increased proteins associated with RNA metabolism and spliceosome pathways, supporting the energy‐splicing resilience axis theory of ageing.

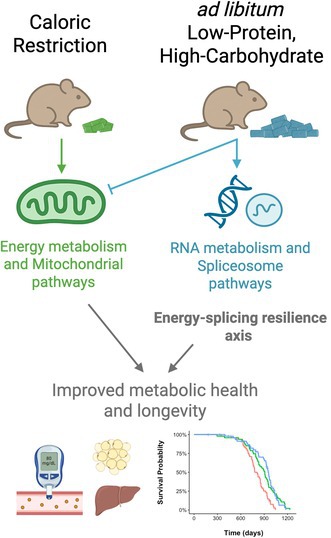

## 1 Introduction

Nutrition has a central role in ageing, influencing longevity, healthspan, age‐related cardiometabolic parameters, and the biological hallmarks of ageing (Green et al. 2022; Ingram and de Cabo 2017; Le Couteur et al. 2024; Lee et al. 2021; Raubenheimer et al. 2022). Caloric restriction (CR) is the most extensively studied dietary approach for delaying ageing and extending lifespan across many taxa. In vertebrates such as mice, rats and nonhuman primates, CR is typically implemented by restricting food intake to 50%–80% of ad libitum‐fed consumption, provided as a single daily portion with micronutrient supplementation (Ingram and de Cabo 2017; Lee et al. 2021). This feeding regimen induces both energy restriction and extended fasting periods because animals usually consume all available food within a few hours and then fast until the next feeding cycle.

The Geometric Framework provides a method for disentangling the complex relationships between phenotypic outcomes and nutritional interventions (Raubenheimer et al. 2022; Simpson and Raubenheimer 2012). In our previous work with ad libitum‐fed mice, we applied this framework and demonstrated that the ratio of dietary protein to carbohydrates influences lifespan (Solon‐Biet et al. 2014). In that study, we incorporated non‐digestible cellulose into certain diets to simulate the effects of CR in ad libitum‐fed animals. This marked the first instance of CR achieved by dietary dilution in mice. As anticipated, mice consuming the high‐cellulose diets increased their food intake as a compensatory response to nutrient dilution, yet their overall energy intake decreased. However, that previous study did not include a comparison to a conventional CR‐treated group. As a result, it could be argued that the longevity benefits observed with ad libitum‐fed low‐protein, high‐carbohydrate diets may not match those from conventional caloric restriction (Couzin‐Frankel 2014; Lee et al. 2021).

Subsequent research indicated that geroprotective effects in mice were more pronounced with standard CR diets that involved fasting, rather than CR achieved through dietary dilution (Pak et al. 2021). It should be noted that this study used dietary dilution with 50% cellulose. We previously found that this high degree of dietary dilution with cellulose reduced lifespan in ad libitum‐fed mice, whereas a more modest dilution provided evidence of lifespan extension (Solon‐Biet et al. 2014).

A recent nutritional intervention that may influence growth and longevity is exome‐matching. This involves manipulating dietary protein so that dietary amino acids are at a ratio matched with the exome, thereby meeting (without excess) the predicted requirements for protein translation under physiological conditions (Piper et al. 2017). In *Drosophila*, exome‐matched diets simultaneously optimized growth and reproduction without a negative cost to longevity. In choice experiments, exome‐matched diets were preferred over diets that were not exome‐matched. In mice on a very low protein (6%) ad libitum‐fed diet, exome‐matching optimized growth and reproductive function (Piper et al. 2017; Wu et al. 2024).

**FIGURE 1:**
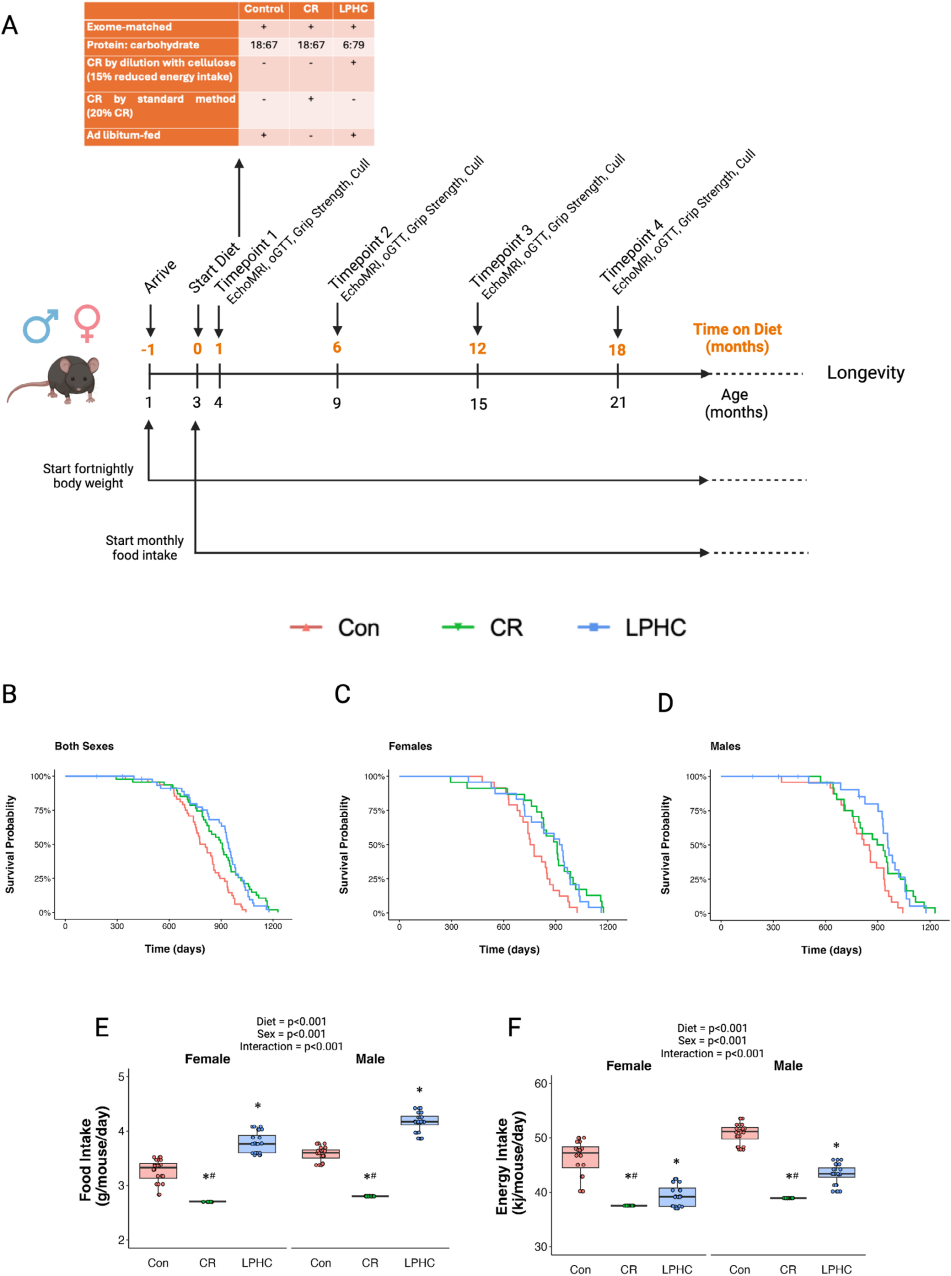
Effects of control, LPHC, or 20% CR diets on lifespan, body weight and intakes of food and energy. Schematic representation of the experimental design (A). Kaplan–Meier survival curves in (B) both sexes combined or separated into (C) males and (D) females. (E) Food and (F) energy intakes of mice calculated as an average of monthly measurements from 6 to 15 months of age. Data presented in E, F expressed as mean ± SEM with box plots also showing individual data points. Statistical analysis was conducted using a two‐way ANOVA and if the ANOVA reached statistical significance (*p* < 0.05) a Tukey posthoc test was performed. Statistical analysis was done in RStudio. Graphing was done in either RStudio or GraphPad Prism. * different to Con; # different to LPHC.

In humans, where voluntary caloric restriction by fasting is unsustainable for most (James et al. 2024), an ad libitum‐fed diet that could generate similar ageing benefits to caloric restriction without periods of fasting has obvious clinical advantages. Here, we compared longevity and ageing in mice on three diets: an ad libitum‐fed control diet (Con); a conventional 20% CR diet; and a low‐protein, high‐carbohydrate (LPHC) ad libitum‐fed diet, which caused caloric restriction through dilution with non‐digestible fibre (Figure 1A, Table S1). The amino acid composition of dietary protein in all diets was exome‐matched (Wu et al. 2024) to reduce variation in food intake caused by an imbalance of amino acids.

## 2 Results

### 2.1 Lifespan, Food and Energy Intake and Body Weight

The composition of the diets is shown in Table S1 and the experimental design in Figure 1A. The Con and CR diets contained 18% protein, 67% carbohydrates, 15% fat (as a percentage of dietary energy content) and ~4% fibre (as a percentage of food weight). The mice on the CR diets received 20% less food than consumed by the mice on the Con diets with ad libitum access to food. The LPHC diet contained 6% protein, 79% carbohydrates and 15% fat and was diluted 30% with non‐digestible fibre (final dilution of ~25% from the Con diet). The protein in all diets was exome‐matched and supplemented with AIN93 trace minerals and vitamins. The diets were commenced at 12 weeks of age.

Survival curves (Figure 1B–D) show that the LPHC and CR diets had similar effects on lifespan compared with the ad libitum control diet. LPHC and CR diets significantly increased median lifespan by 17% and 11%, respectively, compared to the control diet. There was no statistically significant difference between median lifespans of the CR versus LPHC. Maximum lifespans were 1008 days for controls, 1179 days for CR, and 1115 days for LPHC diets. Sex was not a significant effect modifier.

Per protocol, food and energy intakes were reduced by 20% in the CR mice compared to the ad libitum‐fed control mice (Figure 1E,F). Although food intake was increased by 16%–17% with the LPHC diet compared to the control diet, this did not fully offset the dilution by cellulose (i.e., low energy density), and consequently, energy intake was decreased by 15%. This value compares with reported values ranging from 20%–50% reductions in energy intake using conventional CR regimes (Speakman et al. 2016; Swindell 2012). The increase in lifespan in mice on the LPHC diets was not secondary to periods of fasting (Pak et al. 2021) because the mice had ad libitum access to food.

### 2.2 Body Composition and Metabolic Characteristics

**FIGURE 2:**
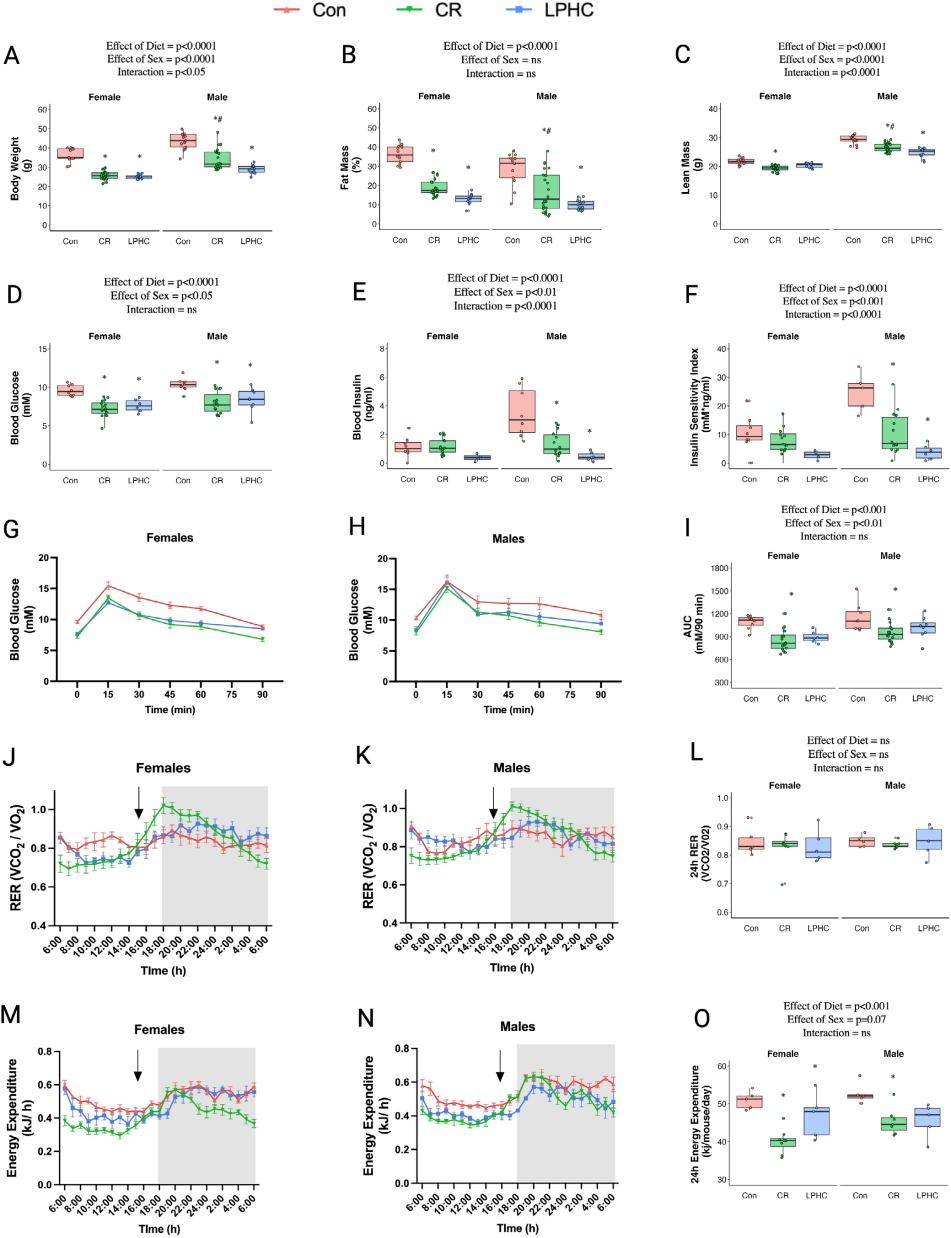
Body composition, blood glucose control and indirect calorimetry at 9 months on diet (12 months of age) of mice fed a control or LPHC diet or were 20% CR. (A) Body weight, (B) fat mass expressed as a percent of body weight, and (C) lean mass of male and female mice fed Con, LPHC or CR. (D) Basal blood glucose and (E) basal blood insulin measured before the GTT, the product of which is used to measure the (F) Insulin sensitivity index, a proxy measure of insulin sensitivity. (G and H) Blood glucose curves from an oGTT in females and males respectively and (I) area under the curve (AUC) for oGTT curves. (J, K) Respiratory exchange ratio (RER) over 24 h in males and females, respectively and (L) average RER over the 24 h period. Energy expenditure in (M) females and (N) males as well as (O) total expenditure over the 24 h period. Black arrow (J, K, M, N) indicates approximately when CR mice were fed. Data presented as mean ± SEM with box plots also showing individual data points. Statistical analysis was conducted using a two‐way ANOVA, and if the ANOVA reached statistical significance (*p* < 0.05), a Tukey posthoc test was performed. Statistical analysis was done in RStudio. Graphing was done in either RStudio or GraphPad Prism. * different to Con.

After 9 months on diet (12 months of age), body weight, percentage body fat and lean mass were highest with the ad libitum‐fed Con diets (Figure 2A–C) and, overall, were higher in males than in females. In males, body fat was lower with LPHC than with CR diets. Blood glucose (Figure 2D) and insulin (Figure 2E) were reduced similarly by both the CR and LPHC diets compared to controls (Figure 2D). Consequently, the insulin sensitivity index was lower (i.e., improved) in mice fed LPHC diets and CR diets (Figure 2F). Although the oral GTT curves were lower in the CR and LPHC diets (Figure 2G,H), the AUC was only significantly different in the CR group (Figure 2I).

RER in the LPHC and Con groups did not show the same robust diurnal range as the CR (i.e., between 1, where carbohydrates are dominating metabolism, and 0.7, where fat is the predominant nutrient source; Figure 2J,K), likely because of the fasting associated with CR. However, the average 24 h RER was not different (Figure 2L). The reduction in energy intake in mice on the CR diet (M 23%, F 19%) approximated the reduction in energy expenditure (M 21%, F 14%) compared to mice on the Con diet. Likewise, the reduction in energy intake in the mice on the LPHC diet (M 15%, F 15%) matched the reduction in energy expenditure (M 13%, F 8%) compared to mice on the Con diet (Figure 2M–O). Of note, the reductions in energy intake and energy expenditure were greater with CR than with the LPHC diet, suggesting that the LPHC is not only acting via reduced energy intake.

**FIGURE 3:**
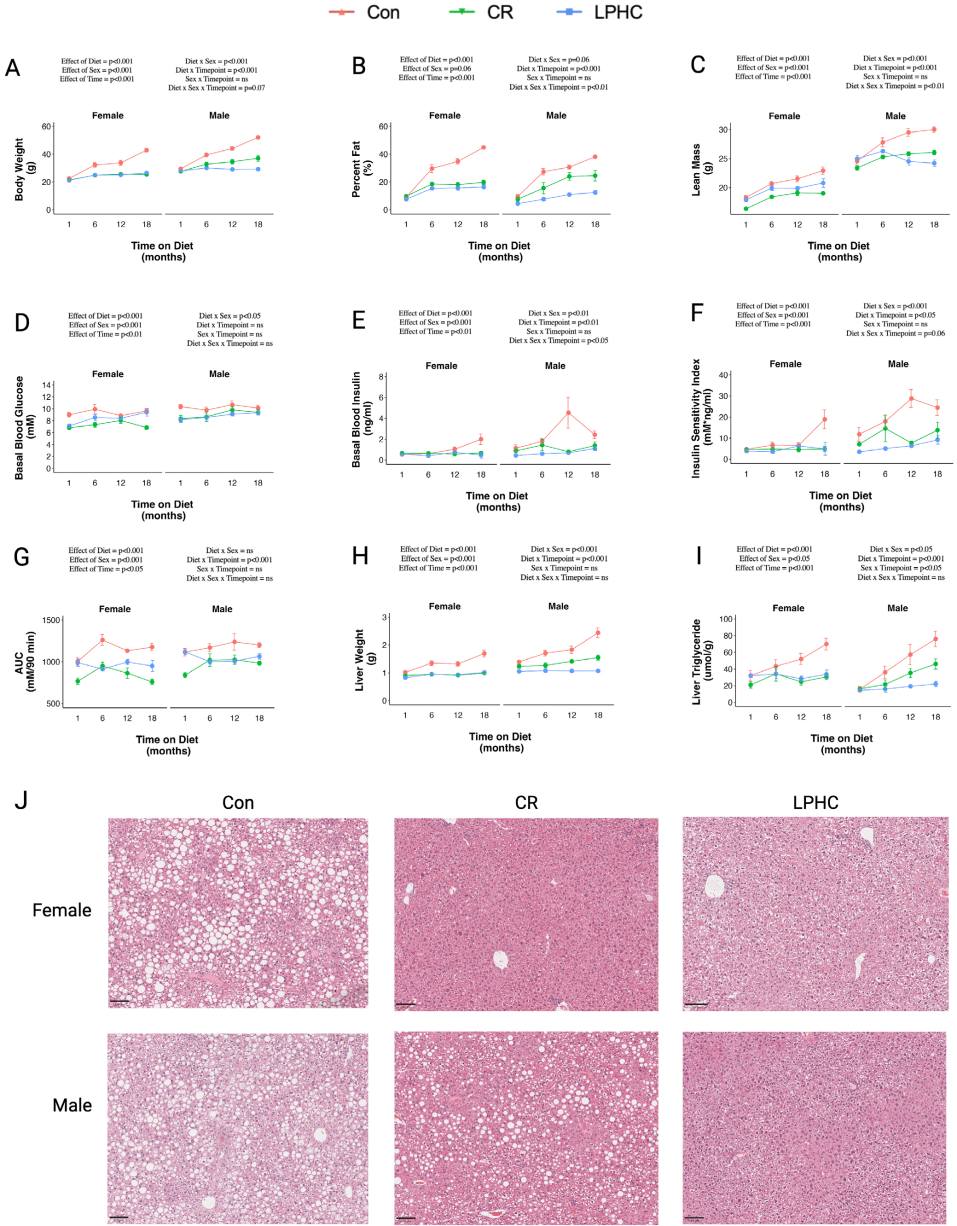
Effects of consuming a control, LPHC or 20% CR diet for 1, 6, 12, or 18 months in male and female mice. (A) Body weight, (B) fat mass expressed as a percent of body weight, and (C) lean mass. (D) Basal blood glucose and (E) basal blood insulin measured before the GTT, the products of which are used to measure the (F) insulin sensitivity index, a proxy measure of insulin sensitivity. (G) Area under the curve (AUC) during an oGTT. Liver (H) weight and (I) triglyceride levels over time. (J) H&E staining of liver at 18 months on diet (21 months of age). Scale bar = 100 mm. Data presented as mean ± SEM with box plots also showing individual data points. Statistical analysis was conducted using a 3‐way ANOVA. Statistical analysis and graphing were done in RStudio.

To determine the age‐related effects on the metabolic responses to diet, a second cohort of mice was culled after 1, 6, 12, or 18 months on the diets (4, 9, 15 and 21 months of age, respectively). Body weight, percentage fat mass and lean mass (Figure 3A–C) increased in control mice with age. Percentage fat mass also increased with age in male CR mice, which has been reported with CR previously (Pak et al. 2021), although the mechanisms remain unexplained. Compared to the control and CR diets, weight and fat mass were relatively stable across age for animals on the LPHC diet.

Glucose and insulin homeostasis deteriorated with age in mice on the Con diet. These outcomes were stable in mice with CR and the LPHC diet (Figure 3D–G), so that at the older ages these were improved compared to the mice on the Con diet. Liver weight and liver triglyceride levels increased with age in the control mice and in males on the CR diet (Figure 3H,I). Histology of the livers was consistent with these results, showing hepatosteatosis in the control mice and male CR mice after 18 months on diets (Figure 3J). At no point for these metabolic, body composition, and liver outcomes did CR outperform the LPHC diets. Indeed, in males, the LPHC diet did better than CR for some parameters.

### 2.3 Liver Protein Abundance and Pathway Analyses

**FIGURE 4:**
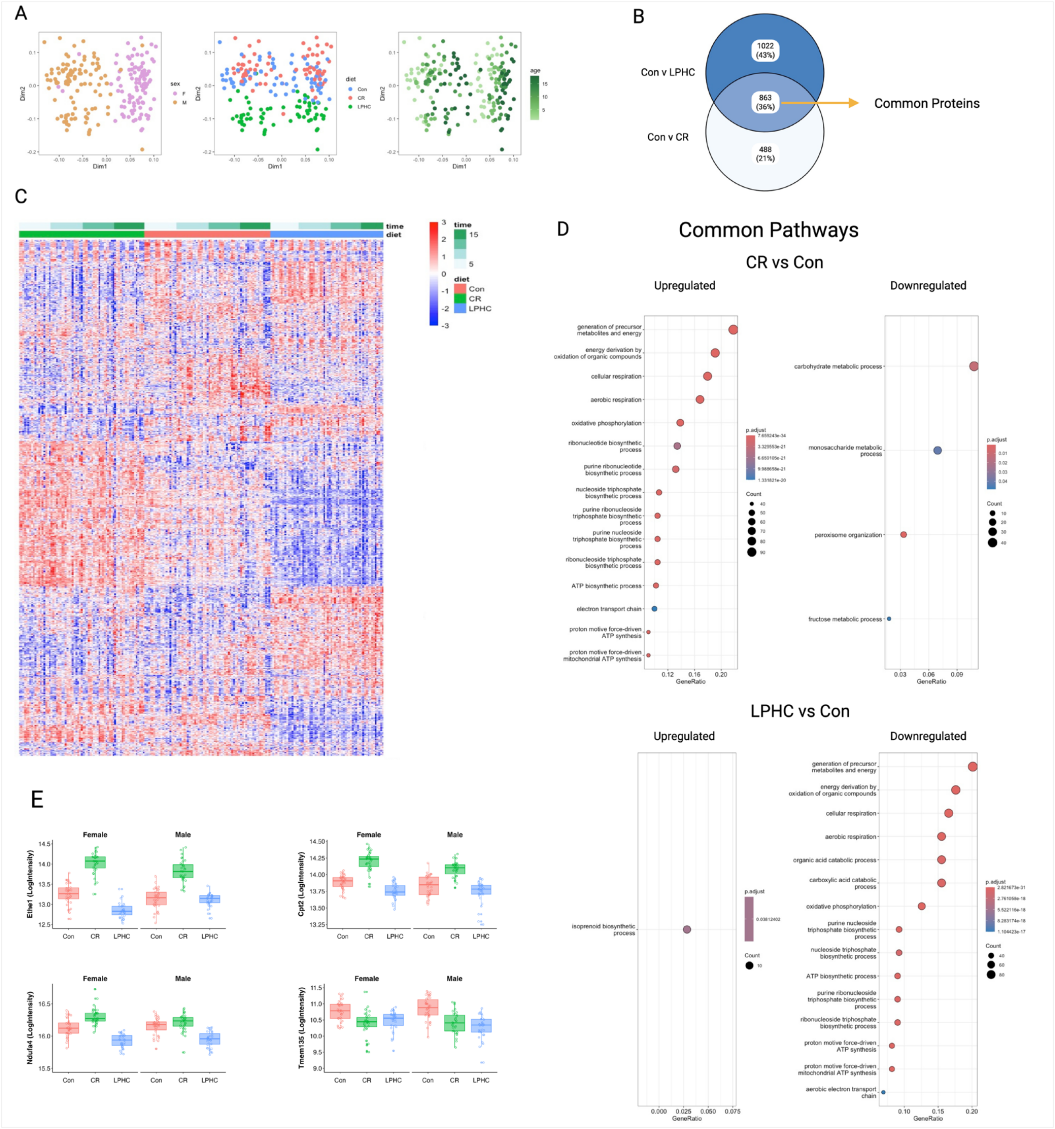
Analysis of proteins that are common to male and female mice fed LPHC or 20% CR diet when compared to Con. (A) Multidimensional scaling (MDS) plot of sex, diet, and age. (B) Venn diagram of pairwise comparisons and overlap of Con vs. LPHC and Con vs. CR with common proteins highlighted. (C) Heatmap of significantly different proteins that are common to LPHC and CR vs. Con‐fed mice. (D) Gene ontology‐biological process (GO‐BP) of common proteins that were up‐ or down‐regulated when compared to Con. (E) Examples (Ethe1, Cpt2, Aass, Ndufa4 and Tmem135) of common proteins determined by the Venn diagram. Significance was determined using the limma package using the following model: Model.matrix(~diet + timepoint + sex + batch); with an FDR of 0.05. Statistical analysis and graphing were done in RStudio.

To determine whether the LPHC and CR dietary interventions act via similar or different cellular mechanisms, the liver proteome was studied in the cohorts of mice culled longitudinally (Figure 4). Multidimensional scaling plots (Figure 4A) indicated that sex, age and diet all influenced the proteome. Therefore, subsequent analyses determining the effects of diet accounted for interactions with sex and age. The Venn diagram (Figure 4B) showed that there were more differentially expressed proteins comparing LPHC to controls (1885 of 3233 proteins detected) than CR versus controls (1351 proteins). There were 863 proteins where the changes in abundances were common between LPHC and CR (Figure 4B; heatmap of common proteins Figure 4C; Top 20 proteins Table S2). There were 1022 proteins unique to LPHC (Top 20 proteins Table S3) and 488 proteins unique to CR (Top 20 proteins Table S4).

The main pathways common to both diets were mitochondrial and related to energy metabolism (Figure 4D). Further analyses of the common proteins shared by CR and LPHC accounted for the possibility that the direction of regulation of protein abundances may be opposite between the two diets. Indeed, these mitochondrial pathways were generally upregulated with the CR diets and down‐regulated with the LPHC diets. Examples of some proteins of interest include mitochondrial proteins (e.g., CPT2, AASS, NSUD4 and TMEM135), where the abundance with LPHC is reduced compared with CR and/or controls (Figure 4E).

**FIGURE 5:**
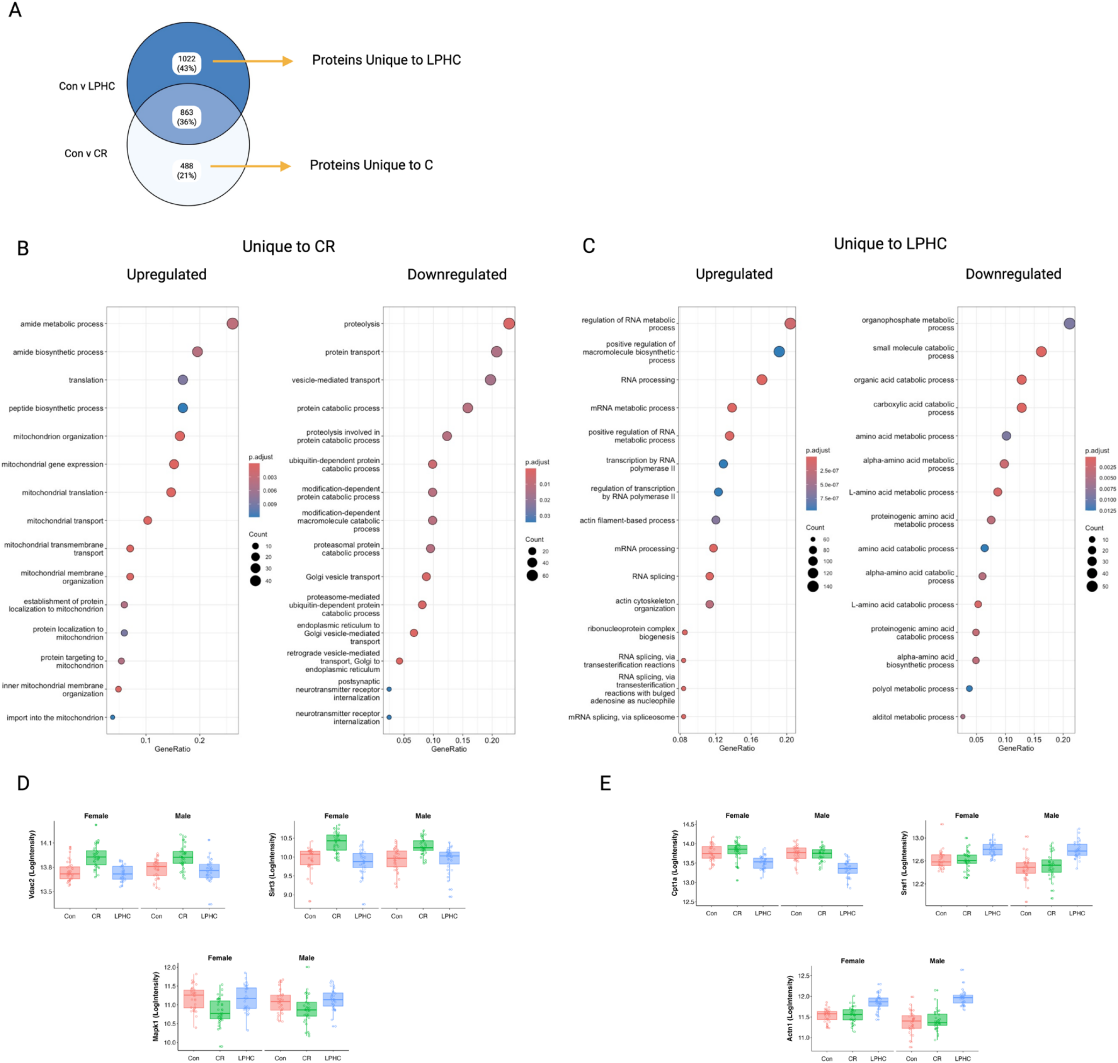
Analysis of proteins that are uniquely different from control in male and female mice fed LPHC or 20% CR diet. (A) Venn diagram of pairwise comparisons and overlap of Con vs. LPHC and Con vs. CR with unique proteins highlighted. (B) Gene ontology‐biological process (GO‐BP) of proteins that were up‐ or down‐regulated in CR mice when compared to control mice. (C) Gene ontology‐biological process (GO‐BP) of proteins that were up‐ or down‐regulated in LPHC mice when compared to control mice. (D) Examples (Vdav2, Sirt3, Mapk1) of proteins unique to CR. (E) Examples (Cpt1, Srsf1, Actn1) of proteins unique to LPHC. Significance was determined using the limma package using the following model: Model.matrix(~diet + timepoint + sex + batch); with an FDR of 0.05. Statistical analysis and graphing were done in RStudio.

Proteins with changes in abundance that were unique to CR included those associated with mitochondrial (upregulated), proteolysis, transport and catabolic (down‐regulated) pathways (Figure 5B,D). Proteins with changes in abundance that were unique to LPHC and upregulated included those involved with RNA metabolism and the spliceosome pathways (Figure 5C,E). This included SRSF1, a key signalling protein for the inverse relationship between cellular energy and the spliceosome (Ferrucci et al. 2022).

## 3 Discussion

Conventional CR, typically involving a 20%–50% reduction in energy intake and protracted fasting intervals between meals, is difficult for humans to sustain, with fewer than one in four participants adherent to such diets in clinical trial settings (James et al. 2024). Here, we found that an ad libitum‐fed diet that was low in protein, high in carbohydrate, and diluted with non‐digestible cellulose (LPHC diet) led to increases in median, mean and maximum lifespan that were comparable to those achieved with conventional 20% CR with periods of fasting.

In the CR group, mice were provided with 20% less food than their ad libitum‐fed controls, given in a single daily portion that was rapidly consumed, thereby inducing extended fasting periods. This was associated with similar reductions in energy intake and energy expenditure (19%–23%). The LPHC diet led to a 15% reduction in energy intake despite an overall increase in total food consumption, as the added bulk via cellulose dilution limited the energy density of the diet. This was associated with a reduction in energy expenditure of 8%–13%. These differences between CR and LPHC diets are modest but suggest that the LPHC is not only acting by reducing energy intake, which was confirmed by the liver proteomics results. The LPHC diet yielded lifespan and metabolic benefits comparable to those of CR, including reductions in body weight, and this was achieved without enforced periods of fasting. The LPHC diet had positive effects on insulin and glucose metabolism, as well as on hepatosteatosis. These characteristics make this dietary approach highly promising if it proves translatable to human populations.

There has been ongoing controversy over whether nutritional modulation of ageing and longevity extension is, or can be, mediated by factors including: (1) reduction in energy intake (Lee et al. 2021; Speakman et al. 2016); (2) reduction in protein intake, alone or relative to carbohydrate (P:C) (Solon‐Biet et al. 2014), or intake of specific amino acids (Green et al. 2023; Solon‐Biet et al. 2019); periods of fasting (de Cabo and Mattson 2019; Pak et al. 2021); and circadian influences on the responses to energy intake (Acosta‐Rodriguez et al. 2022). Our study indicates that periods of fasting are not a prerequisite for longevity extension.

The longevity benefits of the LPHC diet in our study could derive from one or more of three factors: (1) reduced protein intake and the low ratio of protein to carbohydrate (P:C); (2) the 15% reduction in calorie intake, albeit that this had the opposite effect on liver proteins associated with mitochondria and energy metabolism compared to traditional CR; and (3) effects of cellulose, such as changes in the composition of the gut microbiome. We have shown previously that the ratio of protein to carbohydrate impacts lifespan independently of caloric intake (Solon‐Biet et al. 2014), and similar results have been reported in flies and other insects where water, not cellulose, has been used as a dietary dilutant under ad libitum feeding (Le Couteur et al. 2016). We have also shown in mice that cellulose dilution impacts the diversity of the gut microbial community and that community structure is impacted by P:C (Holmes et al. 2017). Parsing these effects is an interesting goal for future work.

Liver proteomics revealed significant dietary effects on fundamental cellular processes, including mitochondrial metabolism and RNA splicing. This is consistent with the central role of the liver in regulating the systemic responses to nutrient intake (Le Couteur et al. 2021). In the CR group, there was an increase in the abundance of proteins involved in mitochondrial pathways and energy metabolism, whereas the LPHC diet was associated with a decrease in proteins associated with mitochondrial pathways and energy metabolism. In a previous study, we observed that dietary protein influenced liver proteins associated with mitochondria (Le Couteur et al. 2021), a finding that is supported by this study, because the LPHC diet resulted in a lower protein intake compared to the CR diet.

The LPHC diet was associated with an increase in the abundance of proteins involved in RNA splicing and metabolism, including the splicing factor SRSF1. SRSF1 links cellular stress, including nutritional stressors, to enhanced spliceosome function and the generation of alternative transcripts. The “energy‐splicing resilience axis theory of aging” proposed by Ferrucci and colleagues (Ferrucci et al. 2022) states that resilience to stressors associated with ageing requires increased RNA splicing and the generation of alternative transcripts. They identified SRSF1 as a key signaling factor orchestrating the response to CR, leading to enhanced RNA splicing. Our study agrees with this hypothesis and extends it into the liver, showing that the LPHC diet led to a reduction in mitochondrial proteins, an increase in RNA splicing proteins, elevated protein levels of SRSF1, and extended longevity. Importantly, we found that it was the *combination* of CR through dilution and the LPHC diet, rather than conventional CR alone, that triggered this “resilience to aging” response. This is consistent with the CALORIE study, where SRSF1 was not identified among the CR‐regulated genes in human muscle (Das et al. 2023). Possible explanations for the difference between splicing in the CR and LPHC groups could be through AMPK signaling. This nutrient sensing enzyme, which is activated in CR, is known to phosphorylate SRSF1, which decreases its ability to bind RNA and thus decreases alternative pre‐mRNA processing (Matsumoto et al. 2020). Another line of evidence for this hypothesis is that treatment of cells from a premature aging disorder (HGPS) with metformin, a known activator of AMPK, decreases mRNA levels of SRSF1 and decreases the levels of pathologic alternative spliced lamin A (Egesipe et al. 2016). Thus, this proteomic signature associated with the ad libitum‐fed LPHC diet suggests it may offer additional benefits beyond those of CR by simultaneously modulating the abundance of proteins involved in both mitochondrial and spliceosome pathways.

In conclusion, an ad libitum‐fed LPHC diet with exome matching and dilution with non‐digestible fibre was as effective as CR in terms of longevity. The LPHC diet may have additional metabolic benefits and involve novel mechanisms compared to CR. Although the CR diet led to an increase in mitochondrial proteins, the ad libitum‐fed LPHC diet led to a reduction in mitochondrial pathways and an increase in SRSF1 and RNA splicing. This result provides a nutritional foundation for the energy‐splicing resilience axis theory of ageing and for dietary interventions that act on this axis.

## 4 Materials and Methods

### 4.1 Animals and Husbandry

All experiments performed were approved by the University of Sydney Animal Ethics Committee (protocols 2018/1453 and 2019/1513).

Male and Female C57BL/6J_Ozarc_ mice were obtained from Ozgene (previously named Animal Resources Centre WA, Australia) at 4 weeks of age and housed at the Charles Perkins Centre's Biological Services Unit at the University of Sydney, Australia. All mice were housed three mice per cage, held at room temperature (21°C–23°C) under a 12 h diurnal light/dark cycle, and were not exercised. Animals were maintained on standard chow ad libitum until commencing experimental diets. Cages used cellulose bedding and were changed fortnightly to minimise negative impacts of high frequency changes on cage microenvironments and animal health (Reeb‐Whitaker et al. 2001).

Animal experiments were split into two parts: a Timepoint (*n* = 9 sex/diet/timepoint) and a Longevity study (*n* = 24 sex/diet/timepoint). At 12 weeks of age, mice from both studies were randomly assigned to either a control (Con), a low‐protein, high‐carbohydrate, low‐energy density diet (LPHC), or a 20% calorie‐restricted (20% CR) diet. Dietary compositions are shown in Table S1 and were purchased from Specialty Feeds Ltd. (Perth, Australia). All diets were provided ad libitum, except for the CR group, where animals were fed daily (~3 pm) with a pre‐weighed portion of the control diet, corresponding to 80% of the measured food intake of the control mice.

Body weights were measured fortnightly, and food intake (by cage) was measured monthly. Animals in the timepoint study that reached the ethical endpoint before their scheduled experimental endpoint (8 out of 216 animals in the timepoint study) were euthanised and excluded from the study. Ethical endpoints reached within the longevity study were maintained as data points for lifespan analysis, the exception being mice with a rectal prolapse occurring without a secondary ailment (as per Pak et al. 2021); 10 from 143 animals in the longevity study, as mice are prone to developing this ailment (Pettan‐Brewer and Treuting 2011). These animals were censored as the date of euthanasia and are indicated as vertical dashes in survival curves in Figure 1B–D.

### 4.2 Body Composition

One week prior to the experimental endpoint (1, 6, 12 or 18 months on diet), mice in the Timepoint study were measured for lean tissue, fat mass and fluid by magnetic resonance imaging using an EchoMRI 900 (EchoMRI, TX, USA). Longevity Study animals were assessed for body composition at 9 months on diet only.

### 4.3 Glucose Tolerance Tests and Insulin Measures

Mice (all Timepoint mice and subset of Longevity study) were fasted for 5 h, and basal blood glucose measurements were taken using a handheld glucometer (Accu‐Chek Performa, Roche Diagnostics Australia Pty Ltd) from the tail tip. Animals were gavaged with 25% glucose solution (2 g/kg lean mass), and blood glucose measurements were taken at 15, 30, 45, 60 and 90 min thereafter.

An additional 10 μL blood was taken from the tail at baseline to determine insulin levels using an enzyme‐linked immunosorbent assay (Crystal Chem, USA). The insulin sensitivity index was estimated using the product of fasting glucose and insulin measures.

### 4.4 Whole Body Calorimetry

At 9 months on diet, a subset of Longevity Study animals underwent indirect calorimetry using the Promethion high‐definition respirometry system (Sable Systems, Las Vegas, NV, USA). Mice were singly housed in Promethion cages for 48 h (including 24 h acclimation), and calorimetric data and physical activity were measured and calculated using the software (MetaScreen). Oxygen consumption (VO_2_) and carbon dioxide production (VCO_2_) were measured to determine the respiratory exchange ratio (RER = VCO_2_/VO_2_).

### 4.5 Animal Cull and Tissue Collections

Animals designated for the Timepoint Study were culled at their assigned experimental endpoints of 1, 6, 12 or 18 months on diet (corresponding to 4, 9, 15 and 21 months of age) for tissue harvesting and analysis. Mice were anaesthetised with Lethabarb (60 mg/mL pentobarbitone sodium) by IP injection and killed by exsanguination via cardiac puncture while anaesthetised. Tissue collection was performed for all groups between 09:00 and 13:00. Caloric‐restricted mice were not fed on the day of sacrifice. The mouse cull order was randomised to prevent time bias.

Blood from the cardiac puncture was placed into a 1.5 mL microcentrifuge tube with EDTA, spun at 10,000 rpm, and plasma transferred to another tube before being stored at −80°C. After the liver was dissected out and weighed, a small portion was fixed in 10% neutral buffered formalin for 24 h and transferred to 70% ethanol before being paraffin‐embedded (Garvan Institute Histopathology Facility). The rest of the liver was snap frozen and stored at −80°C.

### 4.6 Triglyceride Assays

Liver samples from the Timepoint Study were analysed for triglyceride content following a simple lipid extraction protocol (Folch et al. 1957). 20–30 mg of powdered tissue was submerged in 4 mL methanol:chloroform (1:2) mixture and vortexed well. Lipids were extracted by incubating overnight on a rotary tube spinner at 4°C. The next day, 2 mL of 0.6% NaCl was added, samples vortexed and then centrifuged at room temperature (2000 rpm, 10 min). The lower phase was transferred to a glass tube and dried down with a nitrogen evaporator until completely dry. The dried sample was then resuspended in 500 μL ethyl alcohol (Sigma Aldrich).

Triglyceride content was quantified against a glycerol standard (Precimat). Samples and standards (10 μL) were added to a 96‐well plate, dried down at 37°C, and 300 μL Triglyceride reagent (Thermo Fisher Scientific) was added to each well. Samples were incubated at 37°C (30 min), and absorbance was read at 490 nm.

### 4.7 Proteomics Sample Preparation and Mass Spectrometry

30–50 mg of frozen powdered tissue, from the liver of all Timepoint Study mice, was weighed out into a 2 mL microfuge tube and lysed in 1 mL of SDC buffer (4% Sodium Deoxycholate, 100 mM TrisHCL, pH 8.0) at 95°C for 10 min on a thermomixer at 1000 rpm, sonicated in a water bath sonicator for 30 min at room temperature and then centrifuged at 18,000 *g* for 10 min at room temperature. The clarified lysate was transferred to a new tube and stored at −30°C. 5 μL of reduction/alkylation mix (50 mM TCEP, 200 mM chloroacetamide in SDC buffer) was added to 20 μL of clarified tissue lysate in a 1 mL 96‐deep well plate and incubated on a thermomixer at 95°C at 1000 rpm for 10 min, and then 75 μL of water was added. Following the addition of 1 μg trypsin, proteins were digested for 16 h at 1000 rpm and 37°C.

Peptides were cleaned using SDB‐RPS stage tips mounted in a custom 96‐well plate centrifuge rack as described (Harney et al. 2019). Peptides were eluted in 100 μL of 12.5 mM NH_4_OH, 80% acetonitrile, dried down in a vacuum centrifuge and resuspended in 40–60 μL of 5% formic acid. Peptides in 5% (v/v) formic acid were directly injected (injection volume 50 μL) onto a 2.1 × 50 mm, 1.9 μm, InfinityLab Poroshell 120 EC‐C18 analytical column (Agilent). Peptides were resolved over a gradient from 9% acetonitrile to 29% acetonitrile over 13.8 min and then from 29% to 42% over 0.6 min with a flow rate of 0.8 mL/min and then 80% acetonitrile for 2 min at 40°C. The Sciex TripleTOF 6600 system was operated using the TurboSpray ion source in scanning SWATH mode with positive polarity. The scanning SWATH DIA method consisted of 51 variable‐width SWATH DIA windows that spanned the Q1 mass range 390–900 Da.

### 4.8 Proteomics Data Analysis

Raw data were processed in DIANN (v 1.81) (Demichev et al. 2020; Messner et al. 2021) with an in silico generated spectral library, standard settings with “high accuracy”, “robust LC” and “MBR” activated. Peptides were searched against a reference mouse proteome (UP000000589) downloaded from www.uniprot.org on 01/11/2022. The resulting unique gene table was read into R (version 4.3.1), and samples with high numbers of missing values were removed (> 40%). Intensity values were log transformed (base 2). To deal with missing values, proteins were sorted into missing at random (MAR) and missing not at random (MNAR) on the basis of whether there was a difference in the percentage (> 30%) of missing values within the main contrasts of sex (M and F) and age (1 month and 18 months). Proteins were filtered out that were present in fewer than 70% of samples unless determined to be MNAR for sex and/or age. Imputation of missing values from proteins determined as MNAR was performed using a left‐censored imputation method (QRILC) from the QFeatures package (https://www.bioconductor.org/packages/release/bioc/html/QFeatures.html). Data were analysed using limma (Ritchie et al. 2015) with the following model (model.matrix(~diet + timepoint + sex + batch)) and settings (eBayes: trend = T, robust = T). *p* values were corrected for multiple comparisons using the standard Benjamini‐Hochberg correction in limma (FDR). MDS plots were generated using the plotMDS() function in limma. Protein overrepresentation analysis was performed using the clusterProfiler package (Yu et al. 2012) on a background of all detected proteins.

### 4.9 Statistical Analysis

The number indicates individual mice, or individual cages, for food intake measurements. Data are expressed as mean ± SEM unless otherwise stated in the figure legend. Apart from proteomic data, as described above, results were analysed by a two‐way or three‐way ANOVA. If the ANOVA reached statistical significance (*p* < 0.05), a Tukey's post hoc test was used. Statistical analysis was performed using RStudio (Version 2024.04.2 + 764). Statistical significance was set at *p* < 0.05.

## Author Contributions

S.J.S., D.G.L.C., D.R., and A.E.B.: conceptualisation; A.E.B., T.P., L.S., C.S.‐P., and A.M.S.: methodology; A.E.B., T.P., L.S., C.S.‐P., S.S., L.P., A.F., and K.S.I.: investigation; S.J.S., D.G.L.C., J.E.G., J.G., and D.R.: funding acquisition; A.E.B. and T.P.: project administration; S.J.S., D.G.L.C., and G.J.C.: supervision; D.G.L.C. and A.E.B.: writing – original draft; S.J.S., J.E.G., J.G., D.R., G.J.C., L.S., C.S.‐P., and A.M.S.: writing – review and editing.

## Ethics Statement

All experiments performed were approved by the University of Sydney Animal Ethics Committee (protocols 2018/1453 and 2019/1513).

## Conflicts of Interest

The authors declare no conflicts of interest.

## Supporting information


**Appendix S1:** acel70269‐sup‐0001‐AppendixS1.docx.

## Data Availability

Data is available upon request.
