# Correction to “An Ad Libitum‐Fed Diet That Matches the Beneficial Lifespan Effects of Caloric Restriction but Acts via Opposite Effects on the Energy‐Splicing Axis”

**DOI:** 10.1111/acel.70442

**Published:** 2026-03-08

Brandon, A.E., Pulpitel, T., Schmitz‐Peiffer, C. et al. (2025), An Ad Libitum‐Fed Diet That Matches the Beneficial Lifespan Effects of Caloric Restriction but Acts via Opposite Effects on the Energy‐Splicing Axis. Aging Cell, 24: e70269. https://doi.org/10.1111/acel.70269.

In the acknowledgements, we would like to add the following statements:

“The authors acknowledge the provision of computing resources on the National Computational Infrastructure (NCI) by the Australian BioCommons Leadership Share (ABLeS) program. The authors also acknowledge the Sydney Informatics Hub, Australian BioCommons, and NCI support staff for developing the codebase for running DIA‐NN at scale.”

We apologize for this omission.

