# Supplementary material for: An Ad Libitum‐Fed Diet That Matches the Beneficial Lifespan Effects of Caloric Restriction but Acts via Opposite Effects on the Energy‐Splicing Axis
Source: Aging Cell. 2025 Oct 21;24(12):e70269. doi: 10.1111/acel.70269 (PMC12686583; doi:10.1111/acel.70269)
Supplement: Supplementary file 1 — Appendix S1: acel70269‐sup‐0001‐AppendixS1.docx. [file ACEL-24-e70269-s001.docx]

**An ad libitum-fed diet that matches the beneficial lifespan effects of caloric restriction but acts via opposite effects on the energy-splicing axis**

Amanda E. Brandon^1,2,3^*, Tamara Pulpitel^1,2^, Carsten Schmitz-Peiffer^1,2^, Lewin Small^1,2^, Alistair M. Senior^1,2^, Sophie Stonehouse^1,2^, Letisha Prescott^1,2^, Alyssa Face^1,2^, K. Saiful Islam^1,2^, Jenny E. Gunton^4,5^, Jacob George^6^, David Raubenheimer^1,2^, Gregory J. Cooney^1,2^, David G. Le Couteur^1,3,7^* and Stephen J. Simpson^1,2^

^1^Charles Perkins Centre, The University of Sydney, NSW, 2006.

^2^School of Life and Environmental Science, The University of Sydney, NSW, 2006.

^3^ANZAC Research Institute, Concord Hospital and Faculty of Medicine and Health, Sydney, NSW, 2139.

^4^Department of Diabetes and Endocrinology, Westmead Hospital, Sydney, NSW 2145.

^5^Centre for Diabetes, Obesity and Endocrinology Research, Westmead Institute for Medical Research, University of Sydney, Westmead, NSW, 2145.

^6^Storr Liver Centre, Westmead Institute for Medical Research, Westmead Hospital Sydney, NSW, 2145.

^7^School of Medical Sciences, Faculty of Medicine and Health, The University of Sydney, NSW, 2006.

*Corresponding Authors

Amanda E. Brandon – [amanda.brandon@sydney.edu.au](mailto:amanda.brandon@sydney.edu.au)

David G. Le Couteur - [david.lecouteur@sydney.edu.au](mailto:david.lecouteur@sydney.edu.au)

**Supplementary Table 1: Dietary composition of Diets**

| **Ingredient** | **Con and CR** | **LPHC** |
| --- | --- | --- |
|  | g/Kg | g/Kg |
| Protein Mix | 213 | 50.8 |
| Sucrose | 101 | 88 |
| Soybean Oil | 37 | 29.6 |
| Lard | 17.5 | 9.2 |
| Linseed Oil | 5.4 | 4.2 |
| Cellulose Starch | 41 (4%) | 305 (30%) |
| Wheat Starch | 410 | 356 |
| Dextrinised Starch | 134 | 116 |
| Calcium Carbonate | 13.1 |  |
| Sodium Chloride | 2.6 | 2.6 |
| AIN93 Trace Minerals | 1.4 | 1.4 |
| Potassium citrate | 2.5 | 2.5 |
| Potassium Dihydrogen Phosphate | 6.9 | 6.9 |
| Potassium Sulphate | 1.6 | 1.6 |
| Choline Chloride | 2.5 | 2.5 |
| AIN93 Vitamins | 10 | 10 |
|  |  |  |
| **Macronutrient** | **%** | **%** |
| Protein | 18 | 6 |
| Carbohydrate | 67 | 79 |
| Fat | 15 | 15 |
| **Energy Density** | 14.2 | 10.4 |

Con and CR diets are similar to standard laboratory chow and AIN93G.

**Supplementary Table 2: Top 20 protein common to CR and LPHC vs Control diets**

| **Control vs CR** | | | | |
| --- | --- | --- | --- | --- |
| **Gene Symbol** | **Protein Name** | **Biological Process** | **logFC** | **adj.P.Val** |
| Ethe1 | **Persulfide dioxygenase ETHE1, mitochondrial** | Hydrogen sulfide metabolic process | 0.757 | 1.13E-37 |
| Aco2 | **Aconitate hydratase, mitochondrial** | Citrate metabolic process | 0.276 | 2.64E-26 |
| Aass | **Alpha-aminoadipic semialdehyde synthase, mitochondrial** | L-lysine catabolic process | 0.486 | 4.31E-25 |
| Cpt2 | **Carnitine O-palmitoyltransferase 2, mitochondrial** | Carnitine metabolic process | 0.280 | 4.31E-25 |
| Acadvl | **Very long-chain specific acyl-CoA dehydrogenase, mitochondrial** | fatty acid catabolic process | 0.295 | 1.19E-24 |
| Hibadh | **3-hydroxyisobutyrate dehydrogenase, mitochondrial** | valine catabolic process | 0.468 | 2.04E-23 |
| Ogdh | **2-oxoglutarate dehydrogenase complex component E1** | 2-oxoglutarate metabolic process | 0.291 | 2.04E-23 |
| Gldc | **Glycine dehydrogenase (decarboxylating), mitochondrial** | glycine catabolic process | 0.339 | 2.48E-22 |
| Dhrs1 | **Dehydrogenase/reductase SDR family member 1** | carbonyl reductase (NADPH) activity | -0.334 | 3.73E-22 |
| Adhfe1 | **Hydroxyacid-oxoacid transhydrogenase, mitochondrial** | lipid metabolic process | 0.319 | 1.25E-19 |
| Mpst | **3-mercaptopyruvate sulfurtransferase** | hydrogen sulfide biosynthetic process | 0.315 | 1.25E-19 |
| Suclg1 | **Succinate--CoA ligase [ADP/GDP-forming] subunit alpha, mitochondrial** | tricarboxylic acid cycle | 0.194 | 1.25E-19 |
| Cps1 | **Carbamoyl-phosphate synthase [ammonia], mitochondrial** | carbamoyl-phosphate synthase (glutamine-hydrolyzing) activity | 0.425 | 4.03E-19 |
| Ech1 | **Delta(3,5)-Delta(2,4)-dienoyl-CoA isomerase, mitochondrial** | fatty acid metabolism | 0.369 | 3.51E-18 |
| Sod2 | **Superoxide dismutase [Mn], mitochondrial** | cellular response to oxidative stress | 0.315 | 3.51E-18 |
| Mdh2 | **Malate dehydrogenase, mitochondrial** | aerobic respiration | 0.195 | 3.51E-18 |
| Idh3b | **Isocitrate dehydrogenase [NAD] subunit, mitochondrial** | tricarboxylic acid cycle | 0.255 | 2.55E-17 |
| Acad10 | **Acyl-CoA dehydrogenase family member 10** | fatty acid beta-oxidation | 0.280 | 3.45E-17 |
| Fabp2 | **Fatty acid-binding protein, intestinal** | fatty acid metabolic process | -0.625 | 4.26E-17 |
| Sdha | **Succinate dehydrogenase [ubiquinone] flavoprotein subunit, mitochondrial** | respiratory electron transport chain | 0.187 | 4.97E-17 |
| **Control vs LPHC** | | | | |
| **Gene Symbol** | **Protein Name** | **Biological Process** | **logFC** | **adj.P.Val** |
| Ass1 | **Argininosuccinate synthase** | L-arginine biosynthetic process | -1.097 | 1.66E-55 |
| Abat | **4-aminobutyrate aminotransferase, mitochondrial** | positive regulation of aspartate secretion | -0.897 | 5.55E-47 |
| Hgd | **Homogentisate 1,2-dioxygenase** | tyrosine catabolic process | -0.614 | 6.44E-44 |
| Oat | **Ornithine aminotransferase, mitochondrial** | L-proline biosynthetic process | -1.582 | 1.55E-42 |
| Arsb | **Arylsulfatase B** | chondroitin sulfate proteoglycan catabolic process | 1.031 | 1.64E-42 |
| Ftcd | **Formimidoyltransferase-cyclodeaminase** | L-histidine catabolic process | -0.451 | 1.44E-40 |
| Pcca | **Propionyl-CoA carboxylase alpha chain, mitochondrial** | lipid catabolic process | -0.415 | 3.41E-40 |
| Aass | **Alpha-aminoadipic semialdehyde synthase, mitochondrial** | L-lysine catabolic process | -0.817 | 5.92E-40 |
| Gstk1 | **Glutathione S-transferase kappa 1** | glutathione metabolic process | -0.645 | 1.19E-39 |
| Slc25a15 | **Mitochondrial ornithine transporter 1** | L-arginine transmembrane transport | -0.686 | 3.60E-38 |
| Fh | **Fumarate hydratase, mitochondrial** | fumarate metabolic process | -0.438 | 1.36E-37 |
| Gaa | **Lysosomal alpha-glucosidase** | lysosome organization | 0.750 | 1.45E-36 |
| Pccb | **Propionyl-CoA carboxylase beta chain, mitochondrial** |  | -0.412 | 1.45E-36 |
| Cps1 | **Carbamoyl-phosphate synthase [ammonia], mitochondrial** | urea cycle | -0.746 | 4.67E-36 |
| Sfxn1 | **Sideroflexin-1** | serine import into mitochondrion | -0.511 | 5.12E-36 |
| Ttc38 | **Tetratricopeptide repeat protein 38** |  | -0.336 | 3.16E-35 |
| Naga | **Alpha-N-acetylgalactosaminidase** | carbohydrate catabolic process | 0.903 | 8.32E-35 |
| Cth | **Cystathionine gamma-lyase** | lipid metabolic process | -0.733 | 8.53E-35 |
| Gcdh | **Glutaryl-CoA dehydrogenase, mitochondrial** | fatty-acyl-CoA biosynthetic process | -0.420 | 1.97E-32 |
| Adhfe1 | **Hydroxyacid-oxoacid transhydrogenase, mitochondrial** | lipid metabolic process | -0.501 | 2.99E-32 |

Biological processes from UniProt (<https://www.uniprot.org/>)

**Supplementary Table 3: Top 20 proteins unique to CR vs Control**

| **Gene Symbol** | **Protein Name** | **Biological Process** | **logFC** | **adj.P.Val** |
| --- | --- | --- | --- | --- |
| Gcat | **2-amino-3-ketobutyrate coenzyme A ligase, mitochondrial** | L-threonine catabolic process to glycine | 0.474 | 4.03E-19 |
| Etfa | **Electron transfer flavoprotein subunit alpha, mitochondrial** | respiratory electron transport chain | 0.233 | 7.54E-19 |
| Acadl | **Long-chain specific acyl-CoA dehydrogenase, mitochondrial** | lipid catabolic process | 0.309 | 3.07E-18 |
| Hspd1 | **60 kDa heat shock protein, mitochondrial** | protein stabilization | 0.246 | 4.62E-18 |
| Suclg2 | **Succinate--CoA ligase [GDP-forming] subunit beta, mitochondrial** | tricarboxylic acid cycle | 0.193 | 1.96E-17 |
| Hoga1 | **4-hydroxy-2-oxoglutarate aldolase, mitochondrial** | glyoxylate catabolic process | 0.261 | 2.20E-17 |
| Gstt2 | **Glutathione S-transferase theta-2** | glutathione metabolic process | 0.414 | 1.16E-16 |
| Prdm1 | **PR domain zinc finger protein 1** | gene expression | 0.257 | 3.63E-16 |
| Sirt3 | **NAD-dependent protein deacetylase sirtuin-3** | protein deacetylation | 0.413 | 3.63E-16 |
| Rai2 | **Retinoic acid-induced protein 2** |  | 0.296 | 1.06E-15 |
| Vcp | **Transitional endoplasmic reticulum ATPase** | DNA repair | -0.143 | 1.21E-15 |
| Mttp | **Microsomal triglyceride transfer protein large subunit** | lipid metabolic process | -0.257 | 7.06E-15 |
| Acaa2 | **3-ketoacyl-CoA thiolase, mitochondrial** | fatty acid beta-oxidation | 0.232 | 1.96E-14 |
| Acp6 | **Lysophosphatidic acid phosphatase type 6** | lysobisphosphatidic acid metabolic process | 0.240 | 2.97E-14 |
| Timm44 | Mitochondrial import inner membrane translocase subunit TIM44 | protein import into mitochondrial matrix | 0.195 | 4.00E-13 |
| Vdac2 | **Non-selective voltage-gated ion channel VDAC2** | mitochondrial outer membrane permeabilization | 0.163 | 1.27E-12 |
| Gckr | **Glucokinase regulatory protein** | negative regulation of glucokinase activity | -0.280 | 1.12E-11 |
| Dnaja2 | **DnaJ homolog subfamily A member 2** | protein refolding | -0.170 | 2.29E-11 |
| Mlec | **Malectin** | protein N-linked glycosylation | -0.284 | 8.68E-11 |
| Taldo1 | **Transaldolase** | pentose-phosphate shunt | -0.153 | 1.70E-10 |

Biological processes from UniProt (<https://www.uniprot.org/>)

**Supplementary Table 4: Top 20 proteins unique to LPHC vs Control**

| **Gene Symbol** | **Protein Name** | **Biological Process** | **logFC** | **adj.P.Val** |
| --- | --- | --- | --- | --- |
| Got1 | Aspartate aminotransferase, cytoplasmic | aspartate metabolic process | -1.330 | 1.08E-60 |
| Asl | Argininosuccinate lyase | amino acid metabolic process | -0.640 | 4.23E-53 |
| Csad | Cysteine sulfinic acid decarboxylase | L-cysteine catabolic process to taurine | 2.142 | 1.27E-42 |
| Sds | L-serine dehydratase/L-threonine deaminase | lipid metabolic process | -1.199 | 4.10E-41 |
| Gpt2 | Alanine aminotransferase 2 | L-alanine metabolic process | -0.854 | 1.40E-38 |
| Agxt2 | Alanine--glyoxylate aminotransferase 2, mitochondrial | glyoxylate catabolic process | -0.562 | 5.12E-35 |
| Arg1 | **Arginase-1** | arginine metabolic process | -0.465 | 5.12E-35 |
| Aldh1l1 | **Cytosolic 10-formyltetrahydrofolate dehydrogenase** | biosynthetic process | -0.483 | 9.46E-33 |
| Gclm | **Glutamate--cysteine ligase regulatory subunit** | response to oxidative stress | 0.477 | 1.08E-31 |
| Agxt | **Alanine--glyoxylate aminotransferase** | pyruvate biosynthetic process | -0.848 | 2.41E-29 |
| Prodh | **Proline dehydrogenase 1, mitochondrial** | proline catabolic process to glutamate | -0.604 | 7.82E-28 |
| Lmna | **Prelamin-A/C** | nucleus organization | 0.344 | 9.09E-28 |
| Actn1 | **Alpha-actinin-1** | actin filament network formation | 0.440 | 3.73E-27 |
| Kyat1 | **Kynurenine--oxoglutarate transaminase 1** | kynurenine metabolic process | 0.733 | 3.93E-27 |
| Dhtkd1 | **2-oxoadipate dehydrogenase complex component E1** | glycolytic process | -0.751 | 1.36E-26 |
| Rrbp1 | **Ribosome-binding protein 1** | protein transport | 0.334 | 2.48E-26 |
| Snu13 | **NHP2-like protein 1** | ribosomal small subunit biogenesis | 0.453 | 2.72E-26 |
| Apex1 | **DNA repair nuclease/redox regulator APEX1** | DNA repair | 0.471 | 5.48E-26 |
| Otc | **Ornithine transcarbamylase, mitochondrial** | urea cycle | -0.365 | 1.85E-25 |
| Gstt3 | **Glutathione S-transferase theta-3** | glutathione metabolic process | -0.951 | 4.18E-25 |

Biological processes from UniProt (<https://www.uniprot.org/>)
